# Intensive Speech Therapy Programme Combined with a Speech Bulb Prosthesis in the Prosthodontic Rehabilitation of Velopharyngeal Dysfunction

**DOI:** 10.7759/cureus.6951

**Published:** 2020-02-11

**Authors:** Nayana Paul, Cimmy Augustine, Urvashi A Sharma, Kumar Nishant, Shivangini Jyotsna

**Affiliations:** 1 Prosthodontics and Crown & Bridge, Buddha Institute of Dental Sciences and Hospital, Patna, IND; 2 Prosthodontics and Crown & Bridge, Care Dental Clinic, Kannur, IND; 3 Prosthodontics and Crown & Bridge, JSS Dental College and Hospital, Mysuru, IND

**Keywords:** velopharyngeal deficiency, speech bulb prosthesis, speech therapy

## Abstract

Velopharyngeal insufficiency resulting from a defect in the soft palate, acquired or congenital, causes incomplete closure of the palatopharyngeal sphincter. An individual with such a defect suffers from multiple problems in eating, speaking, breathing, as well as psychological trauma, in society. This case report describes the rehabilitation of a patient with a congenital velopharyngeal defect using a definitive speech bulb obturator and an intensive speech therapy program. The patient underwent speech therapy for a period of three months. A speech and voice assessment was conducted before and after speech therapy. A speech intelligibility test was conducted, and nasalance was measured using a nasometer. Significant improvement in speech, mastication, and velopharyngeal function was achieved after bulb reduction and speech therapy.

## Introduction

Velopharyngeal dysfunction (VPD) is a condition where there is a lack of effective closure between the soft palate and one or more of the pharyngeal walls during swallowing and the production of oral speech sounds. The muscles that play an important role in velopharyngeal closure are the Levator veli palatini and the superior constrictor. VPD results in hypernasality, maladaptive articulation errors, and nasal regurgitation. If there is a structural deficiency, it is called velopharyngeal deficiency and if a primary functional cause is suspected, the condition is called velopharyngeal mislearning [1]. The treatment of VPD caused by velopharyngeal insufficiency is surgical with procedures such as pharyngeal flap and sphincter pharyngoplasty [1]. These surgeries result in a reduction of the nasopharyngeal gap to improve velopharyngeal (VP) closure while still allowing for adequate nasal breathing. However, patients may continue to demonstrate hypernasality after a secondary velopharyngeal surgery. The success of these procedures depends as much on the amount and placement of the surgically mobilized tissue as on the ability of the velum and lateral pharyngeal walls to approximate and close any residual openings for speech production [2]. However, not all patients will improve their hypernasal speech sufficiently [3]. The speech prosthesis may be the best choice in several situations where surgery is not the treatment of choice due to systemic, anatomic, or functional limitations or if the patient is not willing to undergo any surgical procedure. Another treatment approach for hypernasality is prosthodontic treatment with a palatal lift or speech bulb prostheses [4-5]. Palatal lifts are typically used with individuals who have a sufficiently long velum that does not move (for example, resulting from a traumatic brain injury or stroke). Speech bulb prostheses are typically used with patients who have structural velopharyngeal deficits. A pharyngeal obturator or “speech bulb” is a removable maxillary prosthesis with an extension protruding into the pharynx. This protrusion separates the oropharynx and the nasopharynx during speaking and swallowing, aiming to improve function, speech, and, ultimately, quality of life for the patient. The advantages of speech bulbs/palatal lifts, when compared to surgical intervention, are less invasive, less expensive, and easier to modify and adjust postoperatively. In patients with a congenital velopharyngeal insufficiency, velopharyngeal insufficiency and mislearning may both be present. Therefore, a combination of both physical (surgery or prosthesis) and functional (speech therapy) treatments may be necessary to achieve optimum outcomes [6-8].

A number of studies have described the concurrent use of speech bulb prostheses and speech therapy and that speech therapy can lead to better outcomes when it is intensive [9]. Blakeley was the first to describe how the use of a speech bulb had led to improve velopharyngeal closure in a patient with a cleft palate [10]. Some studies have shown that a combination of a bulb reduction program and speech therapy can cause a significant improvement in articulation. In some studies, an intensive speech therapy program and careful reduction of the bulb had improved the activity of the velopharyngeal sphincter to such a degree that the patient was able to discontinue the prosthesis [11]. Various case reports have been published in the past regarding the management of velopharyngeal deficiency but a few have emphasized the role of speech therapy [11-15].

The purpose of this case report is to describe the fabrication of a definitive removable prosthesis for a patient with congenital VPD. It highlights the importance of speech therapy to restore optimal function and improve quality of life.

## Case presentation

**Figure 1:**
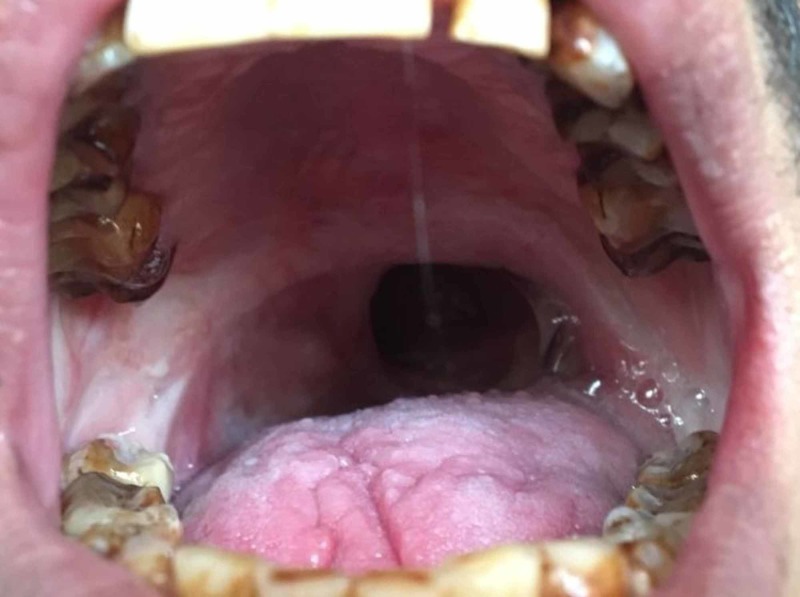
Intraoral view showing the velopharyngeal defect (preoperative)

A 22-year-old patient reported to the Department of Prosthodontics and Crown & Bridge, JSS Dental College and Hospital, Karnataka, India. The patient presented with an extensive defect between the oral and nasal cavity in the soft palate area and impairment in oral function, including speech and swallowing with a nasal twang. He also complained of nasal regurgitation of both liquids and solids. His medical history revealed oral submucous fibrosis on the right and left buccal mucosa. The fibrous bands of submucous fibrosis had been surgically removed. On intraoral examination, a portion of the soft palate and pharynx were missing in the anatomical midline. It extended from the posterior one-third of the soft palate to the middle one-third of the pharyngeal wall, measuring 4 cm in length and 2.5 cm in width (Figure 1). The defect was present since birth. The patient was not willing for surgical correction to improve the nasal regurgitation.

After the clinical evaluation of the defect, it was concluded that the VPD was acceptable for prosthetic rehabilitation. The speech therapist suggested a prosthesis to improve intraoral air pressure and speech intelligibility followed by speech therapy to eliminate compensatory articulation productions. A definitive prosthesis with a hollow acrylic bulb obturator was planned followed by speech therapy. Speech assessment was done without the prosthesis, with the prosthesis, and after the last speech therapy session. The speech was evaluated for nasality with a nasometer. Speech intelligibility was done for high-pressure consonants, low-pressure consonants, and nasals and was assessed using the listener scale. The patient’s consent was taken for the publication of this case report.

Clinical procedure

**Figure 2:**
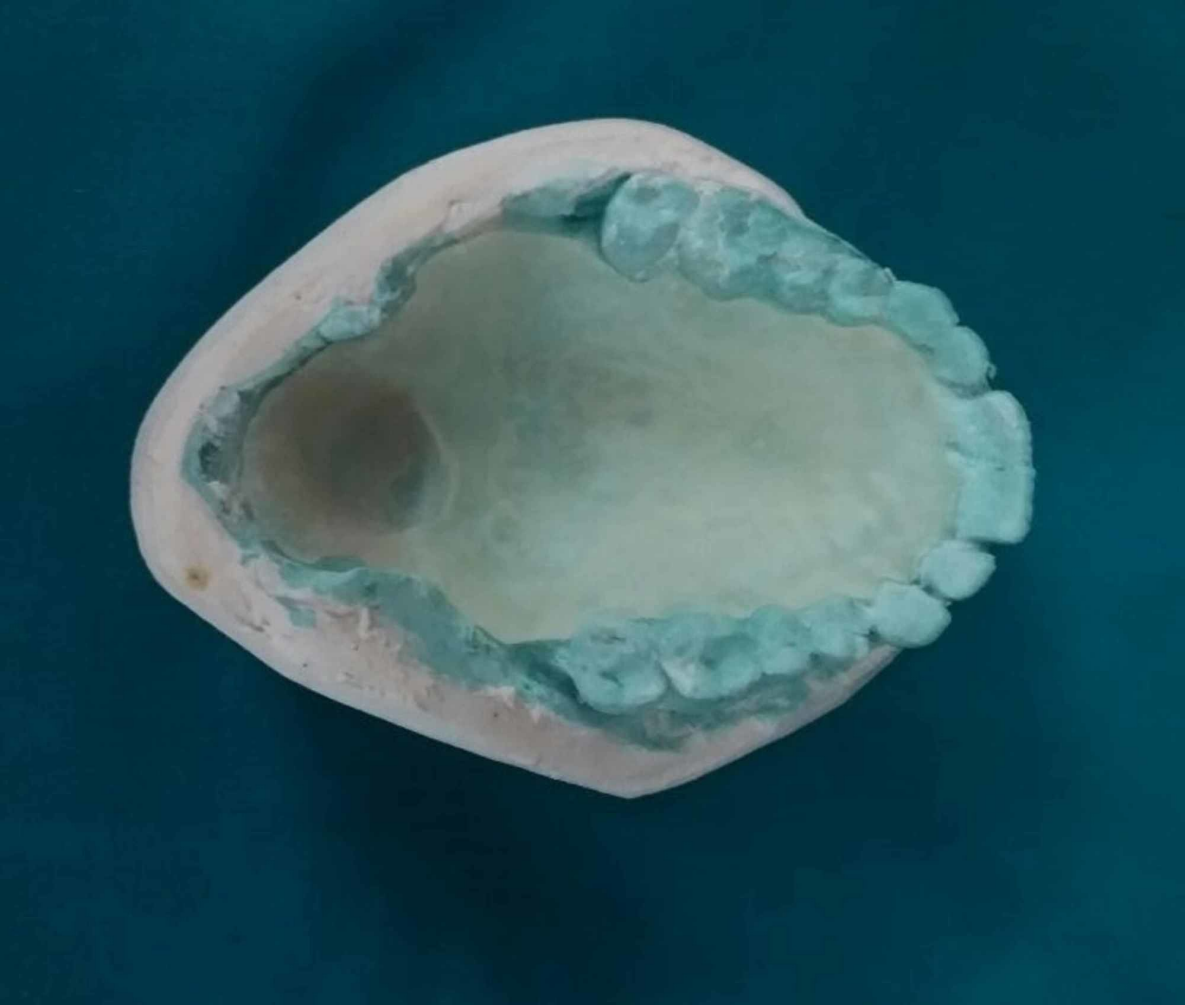
Special tray fabricated using clear acrylic resin

**Figure 3:**
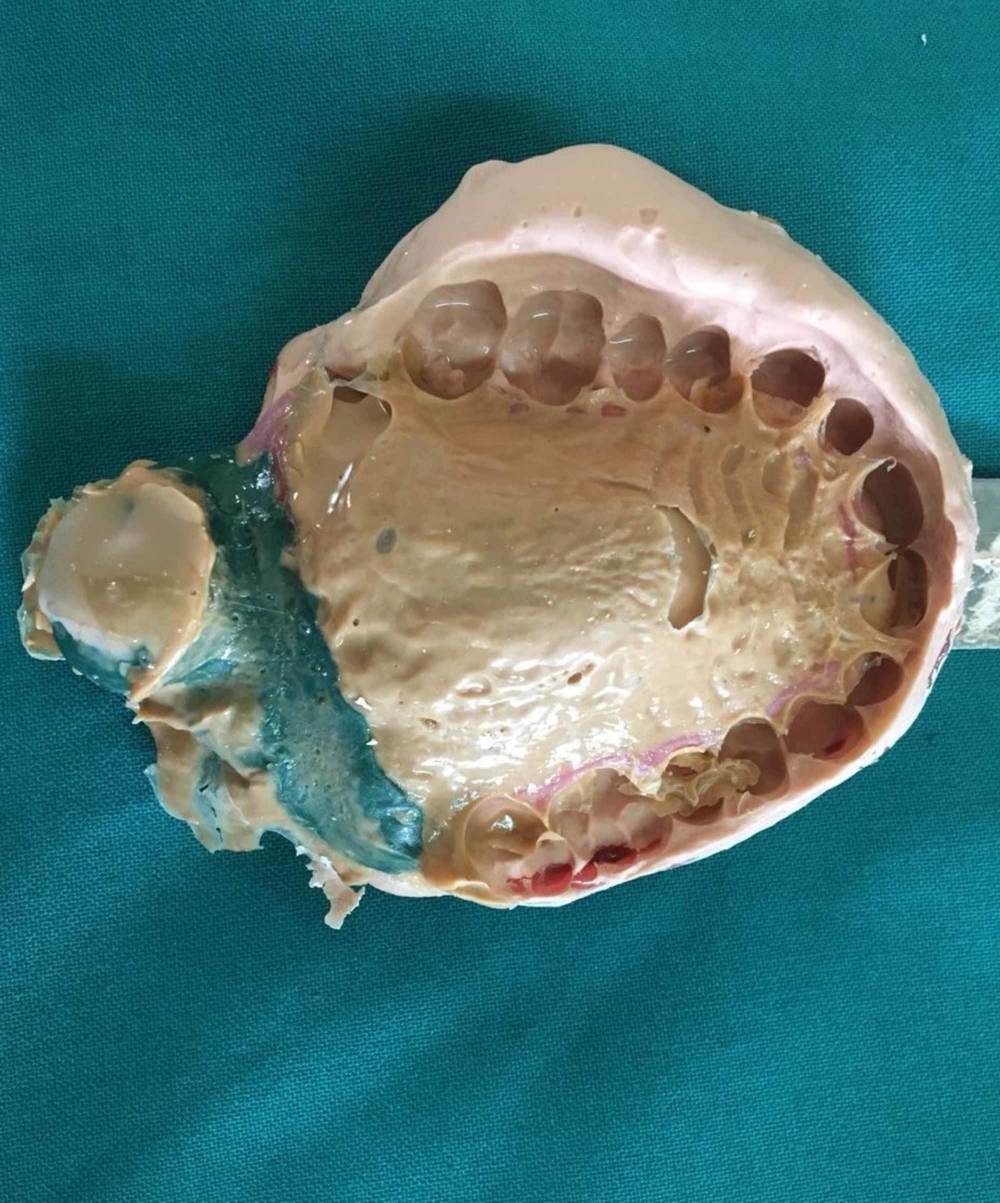
Final impression of the defect

**Figure 4:**
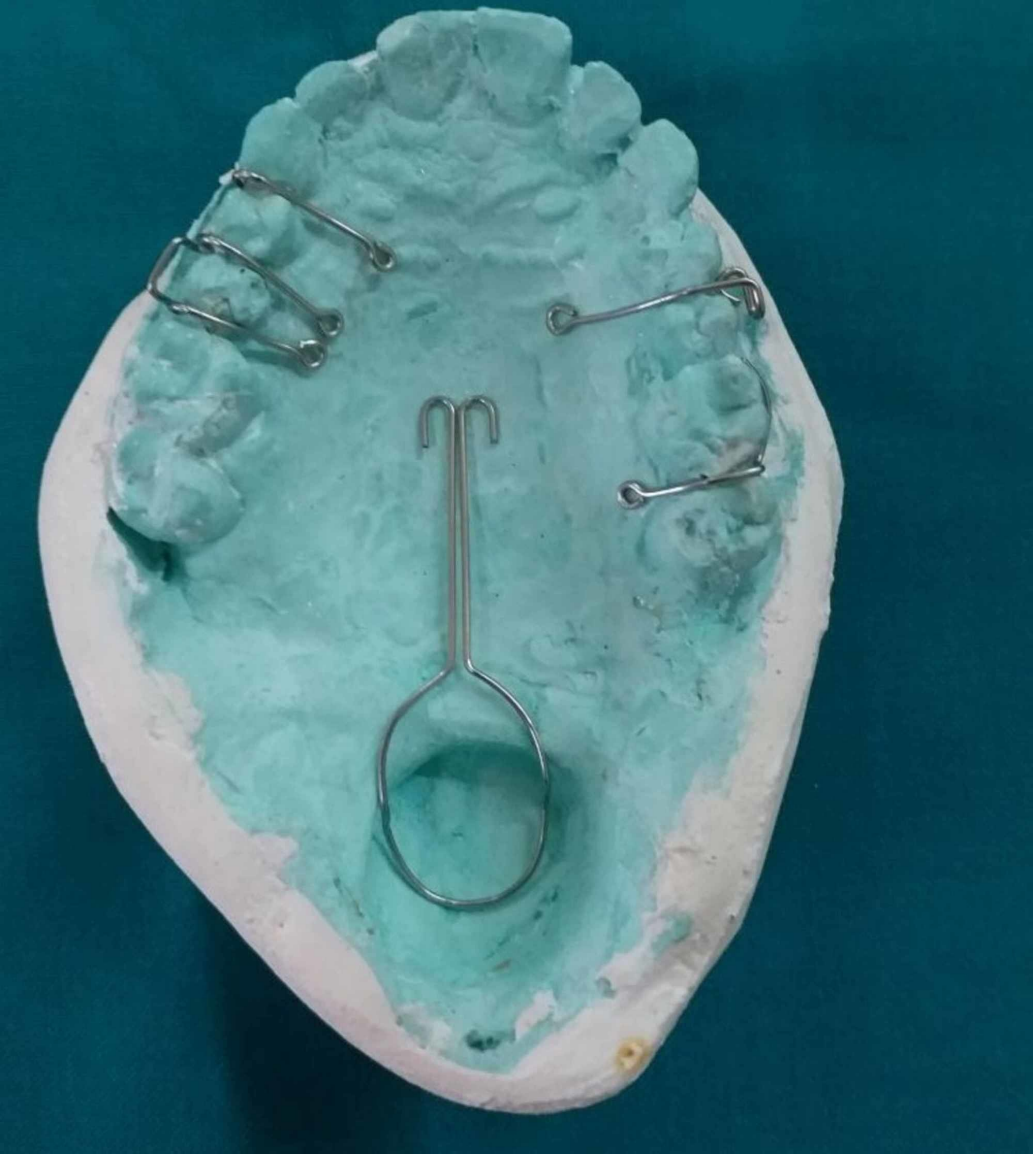
Retentive components adapted to the master cast

**Figure 5:**
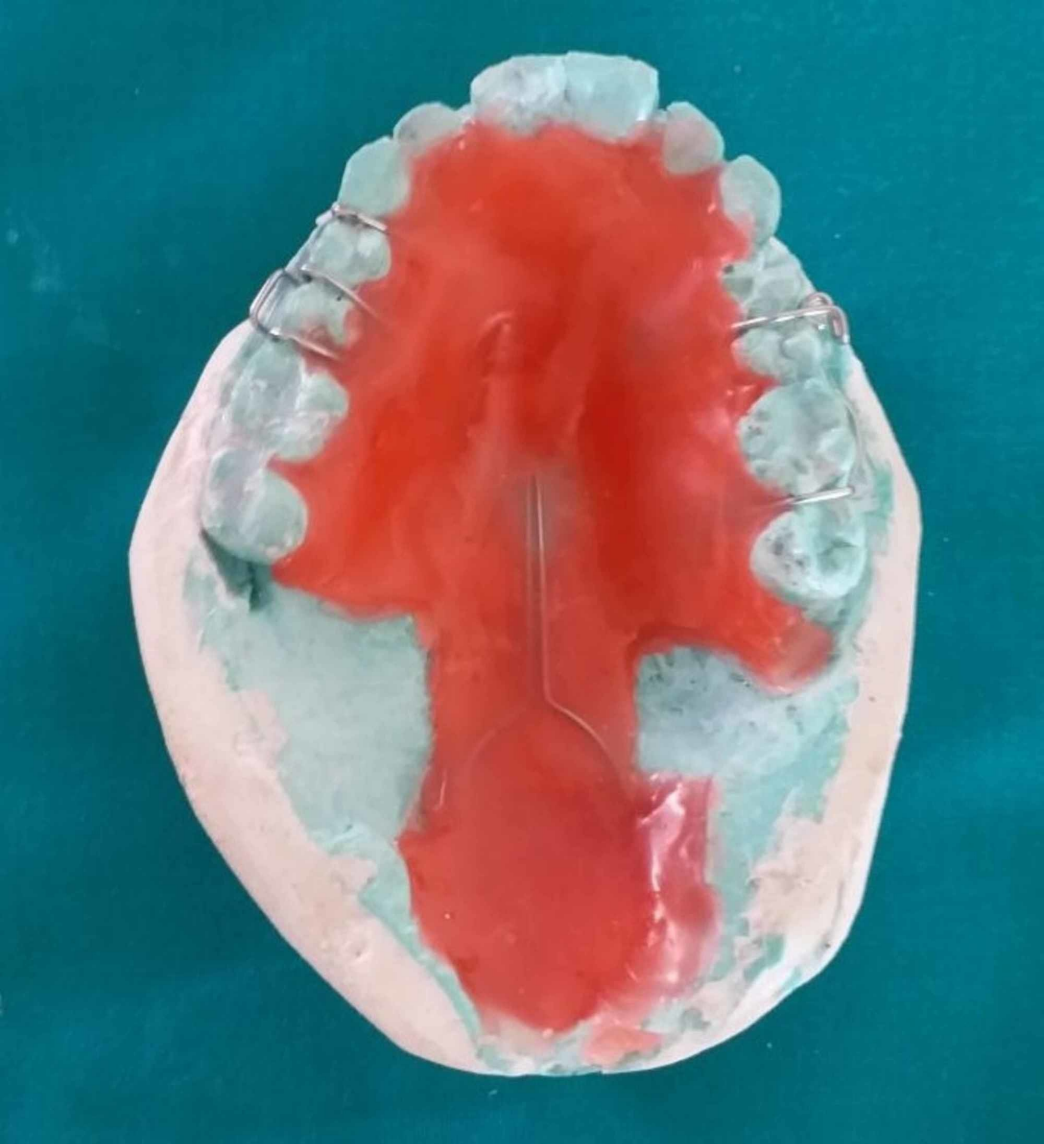
Wax-up

**Figure 6:**
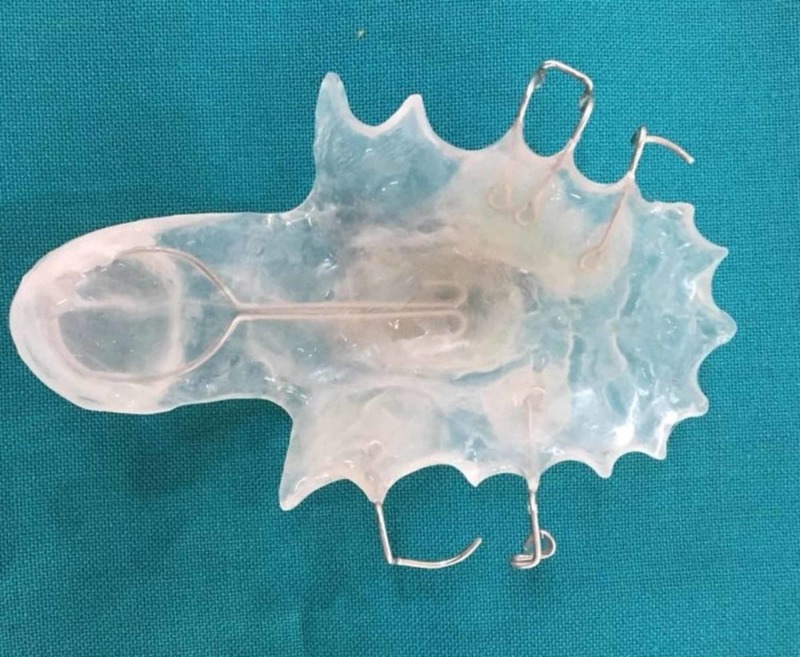
The constructed definitive speech bulb obturator

**Figure 7:**
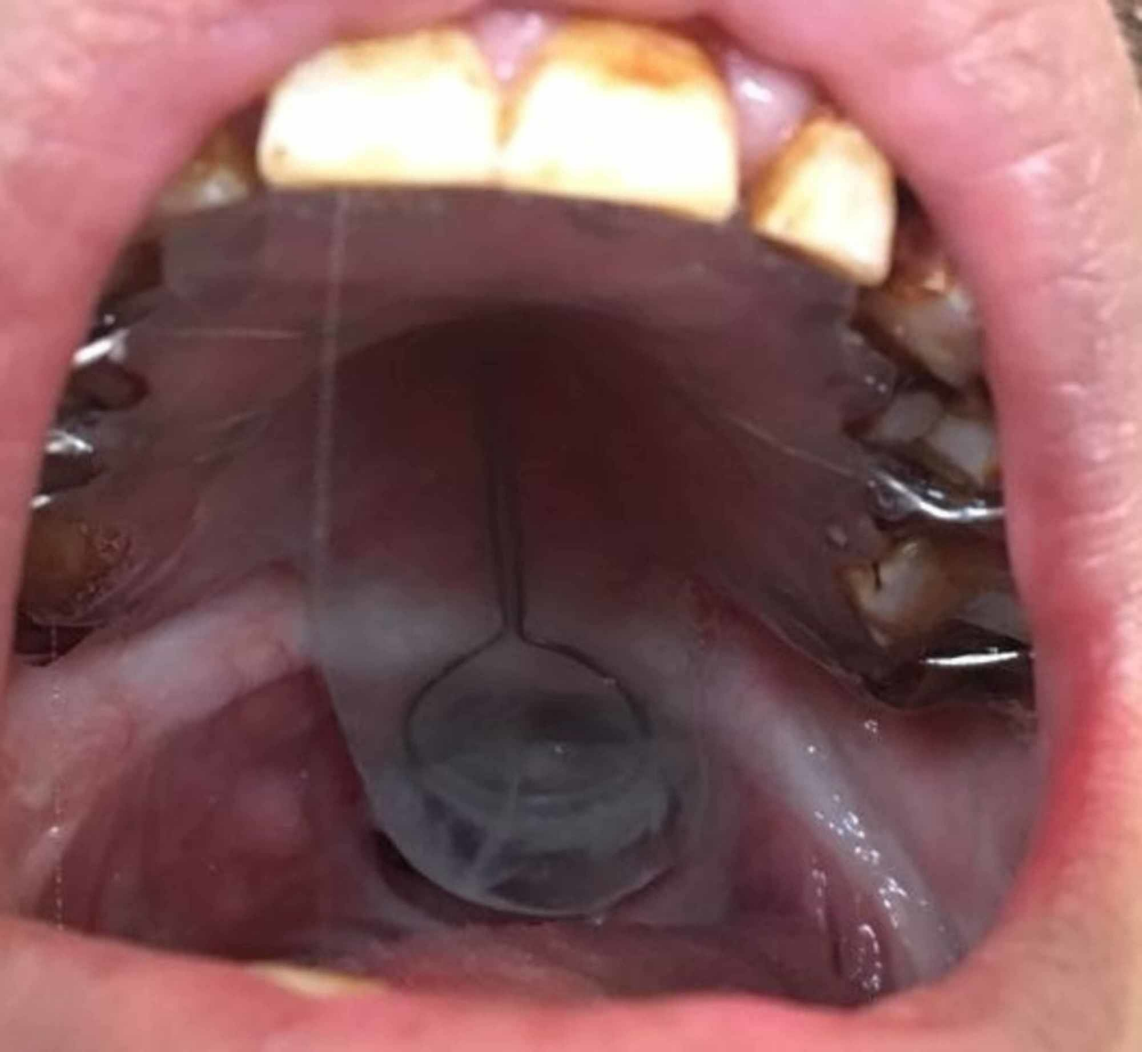
Intraoral view showing the definitive prosthesis in-situ with good approximation of the lateral walls of the “bulb” to the soft tissues that help provide a seal

The soft palatal defect was blocked with a gauze piece and a primary impression of the maxillary arch was made in irreversible hydrocolloid impression material using a stock tray and poured in dental stone. In the retrieved primary cast, the defect was blocked with a gauze piece. In this case report, a dual impression technique has been used. A custom tray was fabricated using an auto-polymerizing acrylic resin (Figure 2). The defect was recorded using a low fusing impression compound. Modification of the bulb was continued until a remarkable reduction in nasal emission was observed. The patient was asked to move his head in a circular manner, from side to side, to swallow and to speak while the wash impression was made in a light body rubber base impression followed by which a pick-up impression was made using irreversible hydrocolloid impression material (Figure 3). The master cast was poured with dental stone. A 21-gauge stainless steel wire was adapted in omega shape to support the pharyngeal part of the prosthesis. Multiple clasps were fabricated in the molars and premolars to provide retention for the prosthesis (Figure 4). Wax-up was completed using modeling wax (Figure 5). Flasking and dewaxing were done, and the mold was packed with a heat-cured clear resin (Dental Products of India, Mumbai, India) and cured. The insertion of the final prosthesis was done and checked for speech, comfort, and retention. Sore spots present on tissues were checked with pressure-indicating paste and relieved. Initially, the patient was having difficulty in breathing. Gradually, the size of the bulb was reduced until the patient felt comfortable. The modified prosthesis and the postoperative picture of the patient is shown in Figure 6 and Figure 7, respectively.

An intensive speech therapy session was recommended for the patient for a period of three months. Speech resonance was measured using a nasometer (Model 6400 II, KayPENTAX Inc., Lincoln Park, New Jersey) at three intervals: before the treatment, with the prosthesis and after completion of an intensive speech therapy program. A speech intelligibility test was also carried out in the local language (Kannada) based on the listener’s scale analysis. Speech samples were recorded in a quiet and soundproof room using a microphone. The patient’s voice was recorded twice, with and without the obturator. SI was calculated using the following formula:

Speech intelligibility (SI) = C/T×100 %

C: Number of words with the correct pronunciation

T: Total number of words in the table

Significant improvement in speech, mastication, and velopharyngeal function was achieved after bulb reduction and speech therapy.

## Discussion

This case report describes the fabrication and subsequent refinement of a speech bulb prosthesis followed by intensive speech therapy. An objective method to determine the treatment option, either surgery or conservative intervention for VPD, was discussed by Shin (Table 1) [16]. According to Shin’s criteria, a severe nasalance score of over 60% should be recommended for surgical intervention for VPD. Based on this criterion and the patient’s willingness, a conservative approach was adopted for the patient. The design of the speech bulb obturator depends on the size and position of the defect and on the surrounding anatomical structures, which can provide support and retention for the prosthesis. The primary goal of the prosthesis was to restore normal VP function, including speaking and swallowing, and to improve the overall quality of life. In the present case, an all-acrylic prosthesis was fabricated with a loop extending into the pharyngeal area for better support. An alternate option is to fabricate a cast partial prosthesis with an acrylic speech bulb. There are certain advantages of an acrylic prosthesis as compared to a cast partial prosthesis. An acrylic prosthesis allows relining and modification and is also cost-effective. Furthermore, using a speech aid can avoid permanent complications of surgical intervention such as snoring, sleep apnea, airway obstruction, and hypo nasality.

**Table 1:** Degree of nasalance and suggested treatment options for VPD (Shin’s criteria)

Nasalance		Recommended treatment options
Below 20%	No nasality	
20-35%	Mild nasality	Speech therapy
35-45%	Moderate nasality	Speech aid appliance with speech therapy
45-60%	High nasality	Surgery or speech aid
Over 60%	Severe nasality	Surgery

In the past, literature has shown a significant improvement in VP function when a combination of a speech bulb prosthesis and speech therapy was used [11,17]. In our case, the patient was subjected to intensive speech therapy for three months. A speech assessment has been done before and after the intervention. There are several methods of speech evaluation, such as acoustic spectrogram, pressure-flow technique, and acoustic and aerodynamic techniques. In the present case, a nasometer was used to detect nasalance before and after the delivery of the prosthesis and 48 hours after the last speech therapy session (Table 2). The biggest improvements to the nasality scores were found after an intensive speech therapy program with the speech bulb in place. This could be interpreted as preliminary evidence that an intensive speech therapy program may improve the effectiveness of a speech bulb prosthesis. The speech intelligibility (SI) test was also conducted before and after the intervention, which was based on the listener’s scale analysis. Three listeners have given their individual scores. The average value of the three listeners’ results was regarded as the SI of the subject. The patient demonstrated improvements in speech intelligibility with the use of a prosthesis, which is in agreement with the results reported in the literature [17-18].

**Table 2:** Mean nasalance scores (%) and standard deviations for the three types of stimuli sentences (high pressure consonants, low pressure consonants, and nasals) without prosthesis and with speech bulb, before and after therapy

	Without Prosthesis	With Prosthesis	After Speech Therapy
High Pressure Consonants	46.65 (7.25)	30.33 (5.33)	13.22 (6.55)
Low Pressure Consonants	47.63 (6.2)	24.25 (4.85)	12.26 (3.9)
Nasals	67.83 (5.33)	64.33 (5.2)	61.06 (6.5)

Although, in our case, a significant improvement has been achieved after bulb modification and speech therapy sessions, literature has shown that the success rate is increased if this intervention is brought about early in life [19]. In grown-ups, when the brain has learned speech, it is more challenging to train the patients using speech therapy. Hence, this article stresses the need to create awareness in the population about VPD and how early treatment can bring about a significant difference in the quality of life of the patient.

## Conclusions

The patient reported more acceptance of the prosthesis after the speech therapy sessions. Reduced hypernasality, improved speech, and improved quality of life have been reported. The present report provides preliminary evidence that an intensive speech therapy program may enhance the effect of a speech bulb to reduce hypernasality in individuals with velopharyngeal dysfunction. However, the effects of the intervention are not uniform in all patients. More research is needed to better understand how patients can derive optimum benefits from a speech bulb prosthesis and a speech therapy program.
